# Factors associated with SARS-COV-2 positivity in nursing workers

**DOI:** 10.1590/1980-220X-REEUSP-2024-0301en

**Published:** 2025-06-06

**Authors:** Jones Sidnei Barbosa de Oliveira, Fernanda Carneiro Mussi, Ana Carla Carvalho Coelho, Jules Ramon Brito Teixeira, Tatiane Araújo dos Santos, Angélica Araújo de Menezes, Luciano de Paula Moura

**Affiliations:** 1 Universidade Federal da Bahia, Salvador, BA, Brazil.

**Keywords:** Nursing, Coronavirus Infections, COVID-19, Pandemics, Surveillance of the Workers Health

## Abstract

**Objective:**

To investigate the association of sociodemographic and occupational variables with SARS-CoV-2 test positivity in nursing workers.

**Method:**

Exploratory and analytical study carried out with a population of 15,936 workers. A secondary database was used and data were analyzed using descriptive and inferential statistics, using the robust Poisson Regression Model and a statistical significance of 5%.

**Results:**

There was predomination of female sex (87.3%), age group from 31 to 59 years (80.3%), brown race/color (63.4%), nursing technicians/assistants (69.6%), role care area (95.1%), outsourced (70.1%) and single (78.2%) employment relation. The age range of 31 to 59 years (95% CI 1.05–1.68) and 18 to 30 years (95% CI 1.12–1.84), black race/color (95% CI 1.02–1.28), having more than one employment relationship (95% CI 1.14–1.33) and having contact with a confirmed case (95% CI 1.03–1.19) were associated with infection in the multivariate analysis.

**Conclusion:**

Detection of infection was associated with adult, black workers, with more than one employment relationship and contact with people infected with SARS-CoV-2.

## INTRODUCTION

The Severe Acute Respiratory Syndrome Coronavirus 2 (SARS-CoV-2) pandemic was rapidly disseminated, prompting the declaration of a Public Health Emergency of International Concern by the World Health Organization^(1)^. Worldwide, more than 760,360,956 cases of COVID-19 and 6,873,477 deaths have been confirmed as of March 2023. During this period, Brazil occupied the 6th place in the ranking of countries with the highest number of cases (37,145,514) and 2nd in number of deaths (699,634), with a fatality rate of 1.9%^(2)^.

Among the population affected by the infection, health workers were largely affected, with 146,685 cases of COVID-19 confirmed in Brazil in 2021 alone^(3)^. According to the US Centers for Disease Control and Prevention, approximately 11% of SARS-CoV-2 infections affected healthcare workers^(4)^. Among them, nursing workers recorded the highest number of cases, with 43,577 in nursing technicians and assistants and 24,719 in nurses. The increased vulnerability to infection for these workers was three times greater than in the general population^(5)^.

The International Council of Nurses and the World Health Organization recorded the existence of 28 million nursing workers in the world, highlighting the relevance of this workforce in health systems. In Brazil, the class represents approximately two million professionals in all organizational structures of the health system and at all levels of care^(6)^. As they constitute the largest healthcare workforce, professionals were on the front lines of the COVID-19 pandemic and due to exposure to the virus, the number of workers infected and requiring hospital care increased^(6)^.

The occupational variables of these workers may be associated with infection. Nurses, nursing technicians and assistants are twenty-four hours in contact with suspected and/or infected patients, working in shifts. In general, they are more exposed to physical fatigue and stress at work^(7,8)^, often assuming more than one job. It is worth noting that the nursing workforce has specificities related to the nature of the work performed, occupational markers (workload, number of jobs, shifts, and intensity of work), social markers (race, gender, social class, and age group), political and economic markers, which can be determinants of greater susceptibility to infection in health services^(9)^.

Despite the differences in health work, there are also differences in the logic and organization of work within categories of nursing workers^(9)^. The nurse performs the care-management work and the nursing assistants and technicians perform the technical-care work, demarcating the technical and social division of labor. Thus, different work processes can provide different types of exposure to illness^(10)^. In this way, the infection cannot be attributed to just one etiological agent without considering the multiple factors influencing it^(11)^ and the heterogeneity of the health workforce.

The above shows that some factors may increase susceptibility of nursing workers to SARS-CoV-2 infection and that the analysis of contamination requires considering the set of synergistic interactions between occupational and sociodemographic issues that increase exposure to infection and illness. The analysis of individual or occupational risk factors is necessary to understand the dynamics of transmissibility, illness and death from infection and to direct rapid and assertive interventions. In this sense, the socioeconomic nature of COVID-19 repositions social aspects in the analysis of the crisis, since policies and programs that reverse social disparities are essential to combat the infection, especially in the context of the work of nursing workers in the Brazilian Public Health System (*SUS*)^(12)^.

However, the analysis of sociodemographic and occupational factors associated with SARS-CoV-2 infection is incipient in the scientific literature for nursing workers compared to studies that indicate associated conditions that can increase the risk of illness in other healthcare professionals^(13)^. Once the factors that may be associated with COVID-19 infection are known, these can be mitigated and analyzed to strengthen public policies and interventions to prevent nosocomial outbreaks and protect nursing workers^(14)^.

It is a fundamental objective of public health to ensure not only the capacity of the health system to provide uninterrupted services to the affected population, but also to prevent the transmission of infection among health professionals, including nursing workers, avoiding physical and mental exhaustion, in addition to cross-infection between colleagues and family members^(15)^. Furthermore, infections among these workers reduce the workforce, increase the workload of those who remain active, and contribute to weakening patient care.

Based on the above, the present study aimed to investigate the association of sociodemographic and occupational variables with the positivity of the SARS-CoV-2 test in nursing workers.

## METHOD

### Design of Study and Local

This is an exploratory and analytical study carried out in the network of assistance institutions and direct and indirect administration of the State Health Department of Bahia (SESAB) linked to the SUS.

### Population and Selection Criteria

The population consisted of 15,936 nursing workers (4,852 nurses and 11,084 nursing technicians and assistants) from SUS SESAB network, who were monitored for the presence of SARS-CoV-2 infection, from March to October 2020.

### Ethical Aspects

The study was approved by the SESAB Ethics Committee, with opinion number 5.380.246 and by the Ethics Committee of the Nursing School of the Federal University of Bahia, opinion number 4.767.147.

### Data Source

A secondary database made available by SESAB, built through monitoring of nursing workers from March 2020, was used. The database consisted of sociodemographic, clinical, occupational variables, performance and results of COVID-19 tests, in addition to data on the occurrence of deaths and hospitalizations of these workers.

### Flow of Monitoring of Nursing Workers from Sesab

The monitoring of nursing workers was carried out by SESAB professionals who are part of the Human Resources Superintendence and the Board of Directors of the Work Management and Health Education (DGTES). A questionnaire was made available to administrative management units, direct and indirect management units, public-private partnerships and interfederative public consortia, and occupational health services under indirect management by the State, to reference professionals and the human resources service of each health unit and to locations where there was no Integrated Worker Health Care Service (SIAST) or Occupational Safety and Monitoring Service.

SIAST and the Occupational Health and Human Resources Sector of each unit were responsible for identifying workers suspected of being infected with SARS-CoV-2 (presence of signs and symptoms and/or contact with a confirmed case); referring workers to undergo testing using reverse transcriptase polymerase chain reaction (RT-PCR) tests and/or antigen testing (rapid test); recording the information in the worker’s medical record and filling out the control spreadsheet for suspected and/or confirmed cases of COVID-19. The data obtained were sent to the Health and Safety Coordination of Healthcare Workers via email for systematization and tabulation in Excel.

**Figure 1:**
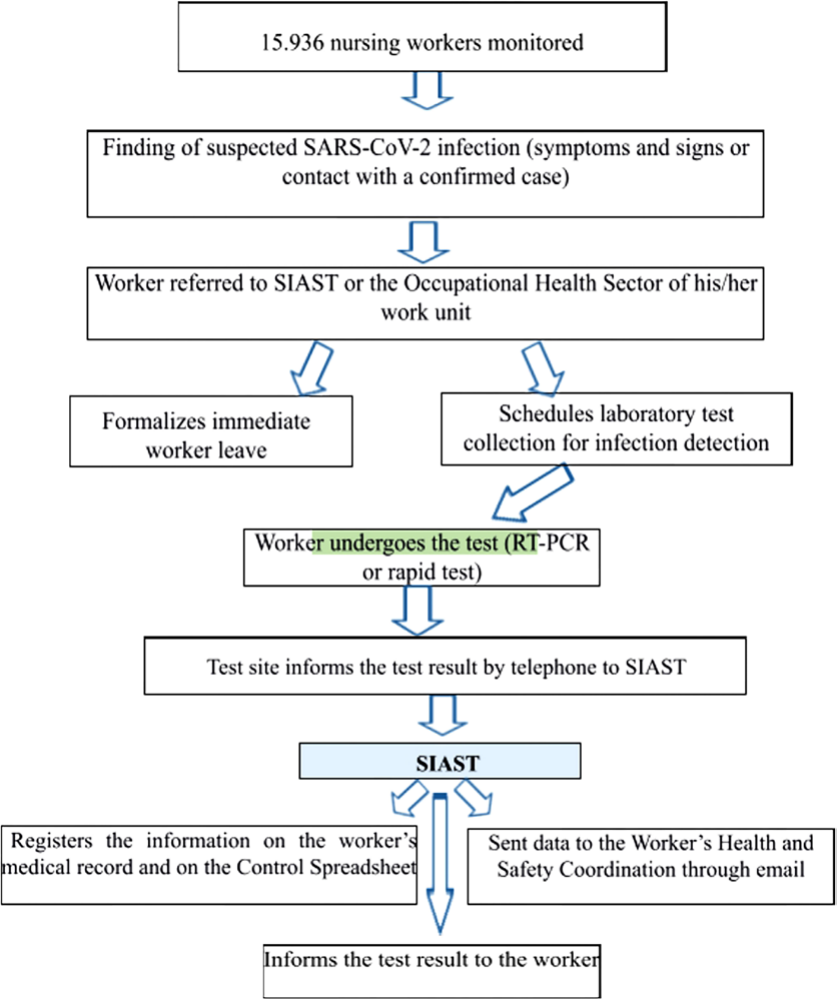
Flow of nursing workers monitoring at the State Health Department of Bahia, Salvador, Bahia, Brazil, 2023.

The flow of nursing workers monitoring and data recording is illustrated in Figure 1.

### Study Variables

The dependent variable of the study was: (0) Infection not confirmed by RT-PCR tests and/or antigen test and (1) Infection confirmed by RT-PCR tests and/or antigen test.

The independent variables were sociodemographic and occupational in nature. Sociodemographic variables included: sex - (0) female, (1) male; age - (0) ≥ 60 years, (1) 31 to 59 years and (2) 18 to 30 years; and race/color - (0) white, yellow and indigenous, (1) brown and (2) black. The occupational variables were related to: professional category - (0) nurses (1) nursing technicians/assistants; type of employment relationship - (0) hired following the Brazilian Public Worker Statute (1) outsourced; number of jobs - (0) One (1) > 1; work sector - (0) management (1) assistance; and contact with a confirmed case of COVID-19 - (0) no (1) yes.

### Data Analysis

Data were tabulated in the *Microsoft Excel* and transported to the *Statistical Package for Social Science* (SPSS), version 21. In the descriptive analyses, absolute and percentage frequencies, means and standard deviations were used. In the bivariate analyses, to verify the association between the independent variables and the outcome, Pearson’s Chi-square test and the detection ratio (DR) and its respective 95% confidence interval were used.

Variables whose associations showed p-value ≤ 0.20 were taken to multivariate analysis using the Poisson Regression Model with robust variance. The backward procedure and the Akaike Information Criterion (AIC) were adopted for choosing the best model. Multicollinearity was ruled out by analyzing the average variance inflation factor of the variables individually, considering a value lower than 10. The statistical significance adopted at this stage was 5%. Data were analyzed in the *Software* Stata, version 15.

## RESULTS

Of the 15,936 nursing workers monitored, 4,057 (25.5%) had a laboratory confirmation of SARS-CoV-2 infection (Table 1).

**Table 1:** Association between sociodemographic and occupational variables and SARS-CoV-2 infection for nursing workers – Salvador, BA, Brazil, 2023.

Variables	Total	Laboratory diagnosis confirmed for SARS-CoV-2			
		Proportion (%)	* *p-value*	^†^ *DR*	^‡^ *95%CI*
**Sociodemographic**					
Age range (n = 14,206)					
≥ 60	423(3.0)	74(17.5)	**0.001**	1.00	–
31 to 59	11,405(80.3)	2,894(25.4)		1.45	1.18–1.79
18 to 30	2,378(16.7)	616(25.9)		1.48	1.19–1.84
Sex (n = 15,936)					
Female	13,908(87.3)	3,513(25.3)	0.131	1.00	–
Male	2,028(12.7)	544(26.8)		1.06	0.98–1.15
Self-declared race/color (n = 11,523)					
White, yellow and indigenous	1,787(15.5)	402(22.5)	**0.030**	1.00	–
Brown	7,310(63.4)	1,691(23.1)		1.03	0.93–1.13
Black	2,426(21.1)	619(25.5)		1.13	1.02–1.26
**Occupational**					
Professional category (n = 15,936)					
Nurse	4,852(30.4)	1.215(25,0)	0,424	1,00	–
Nursing technician and/or assistant	11.084(69,6)	2.842(25,6)		1,02	0,97–1,08
Type of employment relationship (n = 14,317)					
Outsourced	10,030(70.1)	2.644(26,4)	0,248	1,00	–
Permanent contract	4,287(29.9)	1.170(27,3)		0,96	0,91–1,02
Number of jobs (n = 15,890)					
1	12.427(78,2)	2.999(24,1)	**0,000**	1,00	–
> 1	3.463(21,8)	1.055(30,5)		1,26	1,19–1,34
Area of work activity (n = 12,287)					
Managerial	603(4.9)	15(24.9)	0.166	1.00	–
Assistance	11,684(95.1)	3,208(27.5)		1.10	0.96–1.27
Contact with people with COVID-19 (n=15,936)					
No	9,990(62.7)	2,479(24.8)	**0.016**	1.00	–
Yes	5,946(37.3)	1,578(26.5)		1.07	1.01–1.13

*p-value = p-value;

^†^RD = detection ratio;

^‡^CI = 95% confidence interval.

Regarding sociodemographic characteristics, there was a predomination of female sex (87.3%), the age range from 31 to 59 years (80.3%), with an average age of 40.2 years (SD = 9.9), minimum age of 18 years and maximum of 79 years. In terms of self-declared race/color, brown (63.4%) and black (21.1%) workers predominated (Table 1).

Regarding the work context variables, there was a higher percentage of the category of nursing technicians and/or assistants (69.6%). The majority reported an outsourced employment relationship (70.1%) and approximately a quarter (21.8%) had more than one job. Professionals who worked in the healthcare area predominated (95.1%) and more than a third (37.3%) reported previous contact with people diagnosed with COVID-19 (Table 1).

In the bivariate analyses, a higher proportion of infection was found for men (p = 0.131) and in the age group of 18 to 30 years and 31 to 59 years (p-value = 0.001). The infection detection rate increased by 48% and 45%, respectively, for workers aged 18 to 30 years (RD = 1.48; 95% CI = 1.19–1.84) and 31 to 59 years (RD = 1.45; 95% CI = 1.18–1.79) compared to those aged 60 years or older. Workers who self-declared as black were more affected by the infection (p-value = 0.017), with a 13.0% increase in the detection rate for self-declared black workers compared to white, yellow, and indigenous workers (RD = 1.13; 95% CI = 1.02–1.26) (Table 1).

Regarding occupational factors, having had contact with people diagnosed with COVID-19 was associated with infection (p-value = 0.016), with a 1.0% increase observing in the detection rate (RD = 1.07; 95% CI = 1.01–1.13). Furthermore, the proportion of infection was higher for workers with more than one job (p-value = ≤ 0.001), noting a 26% increase (RD = 1.26; 95% CI = 1.19–1.34) in the detection rate. There was no statistically significant association at 5.0% between type of employment relationship, professional category, and area of work (Table 1).

Table 2 presents the multivariate analysis, in which the variables sex, age group, race-color, number of jobs, area of activity and contact with people with COVID-19 were analyzed concomitantly. The infection detection rate was 33.0% higher in the 31 to 59 age group (RD = 1.33; 95% CI = 1.05–1.68) and 44% higher in the 18 to 30 age group (RD = 1.44; 95% CI = 1.12–1.84) when compared to those aged 60 or over. The detection rate increased by 15% in black workers compared to white, yellow and indigenous workers (RD = 1.15; 95% CI = 1.02–1.28). Having more than one job increased the infection detection rate by 23.0% (DR = 1.23; 95%CI = 1.14–1.33), as well as having had previous contact with people with COVID-19 increased the detection rate by 11.0% (DR = 1.11; 95%CI = 1.03–1.19). In the multiple analysis, the model with the lowest Akaike Information Criterion value (AIC = 1.1435) was chosen (Table 2).

**Table 2:** Variables associated with SARS-CoV-2 infection for nursing workers in multivariate analysis – Salvador, BA, Brazil, 2023.

Variables	*DR	^†^95% CI
Age range		
≥ 60 years		
31 to 59 years old	1.33	1.05–1.68
18 to 30 years old	1.44	1.12–1.84
Self-declared race/color		
White, yellow and indigenous		
Brown	1.05	0.95–1.16
Black	1.15	1.02–1.28
Number of jobs		
One		
> 1	1.23	1.14–1.33
Contact with people with COVID-19		
No		
Yes	1.11	1.03–1.19
**AIC**	1.1435	

*DR = detection ratio;

^†^CI = 95% confidence interval.

## DISCUSSION

In this investigation, a significant percentage of nursing workers were infected by SARS-CoV-2. In the same period analyzed, when evaluating workers from different areas of the State Department of Bahia, a higher proportion of infection was observed in nursing workers compared to physiotherapists (18.0%), pharmacists/biochemists (16.3%), doctors (15.8%), psychologists (14.4%), nutritionists (14.0%), and dentists (8.9%). Among the technical level categories, the highest proportions of positive cases occurred in nursing technicians and assistants (22.5%) followed by laboratory or pathology technicians/assistants (22.4%)^(16)^.

Other studies have confirmed a higher incidence of infection among nursing workers. In a survey with 4,854 Iranian healthcare professionals, the incidence of infection was higher in nurses (51.3%) compared to laboratory workers (3.7%), obstetricians (3.0%), radiology technicians (3.0%), and physiotherapists (0.4%)^(17)^. In Wuhan, China, among the 2,457 professionals investigated, the highest incidence was among nurses (52.1%), followed by doctors (33.6%), and pharmacists, laboratory and radiology technicians (14.3%). Furthermore, the highest infection rate was found among healthcare professionals (2.10%) compared to professionals in other areas (0.43%)^(18)^.

Nurses, in general, spend more time in direct contact with patients than other health professionals. During the pandemic, they were on the front line in the fight against the new coronavirus, showing that, due to social marks and history of care, certain bodies were more exposed and more demanded, which made them more vulnerable to the impact of the health crisis^(17)^.

Corroborating the fact that it is not always possible to determine work as the origin of contamination, even with direct contact of professionals with patients suspected or diagnosed with COVID-19, this study demonstrated that sociodemographic and occupational variables were associated with infection, evidencing their influence on disease indicators. Nursing workers were mostly adults, with the multivariate model showing a higher rate of infection detection in younger age groups. Similarly, a study conducted in Southwest Iran with 4,854 health professionals showed that the highest incidence of infection occurred in younger health workers, under 25 years old and between 25 and 45 years old, compared to the general population, and that nurses were the most affected^(17)^.

Research suggests that SARS-CoV-2 infection in younger nursing workers may be associated with inexperience at work, lack of training in the use of personal protective equipment (PPE) and in the management of critically ill patients, which may enhance the chain of viral transmission^(19,20)^. It is possible that greater age is related to increased knowledge and greater experience, favoring greater caution in measures to prevent the transmission of infections and in the adequate use of PPE^(21)^.

In this study, the rate of detection of SARS-CoV-2 infection was higher in self-declared black workers, corroborating an investigation carried out with 14,441 health professionals from the National Health Service in England, in which the highest rate of contamination was found in professionals of BAME (black, Asian, or any other ethnic minority) ethnicity compared to those of white ethnicity^(22)^. The authors considered that higher rates in healthcare professionals from BAME backgrounds may be linked to worse economic and social indicators^(22)^. In this scenario, enhanced surveillance and infection control measures should be a priority in these social groups^(18)^.

According to the report on the socio-racial profile of nursing in Brazil, black women represent 53.0% of nursing workers in the country. Although there is a higher percentage of black nursing technicians and assistants (40%) than nurses (32.5%), both categories tend to occupy more precarious jobs, face various occupational risks and deal with a shortage of PPE^(23,24)^. The fact that black nursing workers make up the most exposed group during the pandemic is in line with historical vulnerabilities of the profession marked by the synergy of social markers of gender, social class, and race-color.

SARS-CoV-2 infection was also associated with having more than one job, corroborating research carried out with 415 nursing professionals at a teaching hospital in the city of São Paulo, which showed a 2.27 times greater chance of infection for those who worked in more than one health service^(25)^. Many nursing workers had more than one job, mainly due to low wages and precarious employment relationships^(26)^. The greater the number of jobs, the greater the time of exposure to risk factors for COVID-19, such as the increased likelihood of contact with infected patients and coworkers, which makes workers more vulnerable to infection and physical and mental illness^(7,13,14)^.

It is important to highlight that the higher viral load to which workers are exposed in the work environment can influence the risk of contamination. A Brazilian ecological study, with 7,201 nursing professionals with COVID-19, showed a higher viral load in the hospital environment, which increased the risk of infection for these professionals. The Global Burden of Disease (GBD) estimated at 0.051 (0.032–0.074) was used as a parameter, which was on an upward trend, with a greater impact on younger people and women^(26)^.

Another factor associated with infection was having contact with a confirmed case, reinforcing that the provision and adequate use of PPE for nursing workers is essential. National and international studies have also found a higher proportion of positive tests for SARS-CoV-2 in healthcare professionals who had direct contact with infected patients, family members, or co-workers^(5,15,27,28)^.

In the present study, there was no association between the nature of the work, either in assistance or managerial, and SARS-CoV-2 infection. Likewise, there was no association between the professional category (nursing technicians/assistants or nurses) and infection. In another investigation, an infection rate of 15% was observed among professionals directly involved in patient care, 16% among those who were not directly involved but worked in high-risk areas such as laboratories, and 18% among nonclinical workers. Thus, participation in direct patient care was not considered a risk factor for positivity in the SARS-CoV-2 RT-PCR test^(29)^. Other studies have also found that exposure to infection risk was higher among administrative staff, suggesting that these professionals may underestimate the risk of contamination because they are not directly involved in patient care, which often leads to non-adherence to mask use. One possible explanation for this is the reduced awareness that infections can also be transmitted by co-workers^(21,30)^.

From the point of view of occupational factors and occupational safety in health, the investigation revealed the need for investment in actions that promote occupational health, ensuring individual and collective protection in the work environment. It also reveals that it is essential to direct attention to professional training for working in pandemic contexts, especially with a focus on younger and less experienced professionals, as well as highlighting the need to consider social markers of risk for illness, such as a greater number of jobs and black race, in infection prevention and control strategies. The study showed the need for economic valorization of work in the field of nursing to minimize job insecurity and allow for a reduction in the number of jobs. Given the association between the factors analyzed, it is possible to consider SARS-CoV-2 infection as an incident and/or injury also related to work^(7,20)^.

This study has some limitations. First, it was carried out with a group of nursing workers from a single state in Brazil. Second, the number of confirmed cases was gathered during the monitoring period of SESAB workers, not covering the entire duration of the pandemic, which may have led to underestimation of infection rates. Finally, possible factors related to indicators of SARS-CoV-2 infection, such as personal hygiene measures and use of PPE, were not considered.

However, the results of this study highlighted and reinforced social and occupational risk factors that demand effective public policy responses against COVID-19, in addition to actions aimed at valuing nursing work. The study also highlighted the need for support and alerts for younger workers, with multiple jobs and those who self-identify as black, who face a higher risk of infection. Furthermore, it reinforced the importance of providing quality PPE and the rigorous adoption of protective measures. Thus, this study provides valuable lessons for managing new health crises.

## CONCLUSION

A significant number of nursing professionals had their laboratory diagnosis confirmed for SARS-CoV-2. The variables that contributed most to infection were age range between 18 and 59 years, black race/color, having more than one employment relationship and having contact with infected people, showing their influence on COVID-19 indicators. These variables highlighted the importance of public policies and interventions aimed at supporting workers, promoting a safe work environment and the economic valorization of nursing work, which involves ensuring decent wages consistent with their responsibilities.

## Data Availability

The dataset supporting the findings of this study is not publicly available.
