# Supplementary material for: Factors associated with SARS-COV-2 positivity in nursing workers
Source: Rev Esc Enferm USP. 2025 Jun 6;59:e20240301. doi: 10.1590/1980-220X-REEUSP-2024-0301en (PMC12147232; doi:10.1590/1980-220X-REEUSP-2024-0301en)
Supplement: Translation

# Translation: Fatores associados à positividade para o SARS-COV-2 em trabalhadores da enfermagem

**DOI:** 10.1590/1980-220X-REEUSP-2024-0301pt

**Authors:** Jones Sidnei Barbosa de Oliveira, Fernanda Carneiro Mussi, Ana Carla Carvalho Coelho, Jules Ramon Brito Teixeira, Tatiane Araújo dos Santos, Angélica Araújo de Menezes, Luciano de Paula Moura

**Affiliations:** 1 Universidade Federal da Bahia, Salvador, BA, Brasil.

**Keywords:** Enfermagem, Infecções por Coronavírus, COVID-19, Pandemias, Vigilância em Saúde do Trabalhador

## Abstract

**Objetivo:**

Investigar a associação de variáveis sociodemográficas e ocupacionais com a positividade do teste para o SARS-CoV-2 em trabalhadores da enfermagem.

**Método:**

Estudo exploratório e analítico realizado com a população de 15.936 trabalhadores. Utilizou-se um banco de dados secundário e os dados foram analisados pela estatística descritiva e inferencial, empregando-se o Modelo de Regressão de Poisson robusto e a significância estatística de 5%.

**Resultados:**

Predominaram o sexo feminino (87,3%), a faixa etária de 31 a 59 anos (80,3%), a raça/cor parda (63,4%), os técnicos/auxiliares em enfermagem (69,6%), a função área assistencial (95,1%), o vínculo terceirizado (70,1%) e único (78,2%). A faixa etária de 31 a 59 anos (IC 95% 1,05–1,68) e de 18 a 30 anos (IC95% 1,12–1,84), a raça/cor preta (IC95% 1,02–1,28), ter mais de um vínculo de trabalho (IC 95% 1,14 –1,33) e ter contato com caso confirmado (IC 95% 1,03–1,19) foram associados a infecção na análise multivariada.

**Conclusão:**

A detecção da infecção foi associada a trabalhadores adultos, negros, com mais de um vínculo e contato com pessoas infectadas pelo SARS-CoV-2.

## INTRODUÇÃO

A pandemia do Coronavírus 2 da Síndrome Respiratória Aguda Grave (SARS-CoV-2) foi rapidamente disseminada, impulsionando a declaração de Emergência em Saúde Pública de Importância Internacional pela Organização Mundial da Saúde^(1)^. No mundo, foram confirmados até março de 2023 mais de 760.360.956 de casos da COVID-19 e 6.873.477 mortes. O Brasil ocupou nesse período a 6ª posição no ranking dos países com maior número de casos (37.145.514) e a 2º em número de mortes (699.634), com letalidade de 1,9%^(2)^.

Entre a população acometida pela infecção, os trabalhadores da saúde foram amplamente afetados, confirmando-se, apenas em 2021, 146.685 casos da COVID-19 no Brasil^(3)^. De acordo com o Centro de Controle e Prevenção de Doenças dos Estados Unidos, cerca de 11% das infecções pelo SARS-CoV-2 acometeram profissionais da saúde^(4)^. Entre eles, os trabalhadores do campo da enfermagem registraram o maior número de casos, com 43.577 em técnicos e auxiliares de enfermagem e 24.719 em enfermeiros. A vulnerabilidade aumentada à infecção para esses trabalhadores foi três vezes maior que na população em geral^(5)^.

O Conselho Internacional de Enfermeiras e da Organização Mundial da Saúde registrou a existência de 28 milhões de trabalhadores da enfermagem no mundo, marcando a relevância dessa força de trabalho nos sistemas de saúde. No Brasil, a classe representa aproximadamente dois milhões de profissionais em todas as estruturas organizacionais do sistema de saúde e em todos os níveis de atenção^(6)^. Por constituirem a maior força de trabalho em saúde, os profissionais estiveram na linha de frente da pandemia da COVID-19 e devido à exposição ao vírus, o número de trabalhadores infectados e que necessitaram de atenção hospitalar aumentou^(6)^.

As variáveis ocupacionais desses trabalhadores podem estar associadas a infecção. Enfermeiros, técnicos e auxiliares em enfermagem estão vinte e quatro horas em contato com pacientes suspeitos e/ou infectados, atuando em regime de turnos. Em geral, estão mais expostos ao cansaço físico e ao estresse no trabalho^(7,8)^, muitas vezes assumindo mais de um vínculo. Destaca-se que a força de trabalho em enfermagem tem especificidades relacionadas à natureza do trabalho desenvolvido, a marcadores ocupacionais (carga horária, número de vínculos, turnos e intensidade do trabalho), sociais (raça, gênero, classe social e grupo etário), políticos e econômicos, os quais podem ser determinantes de maior suscetibilidade a infecção nos serviços de saúde^(9)^.

Não obstante as diferenças no trabalho em saúde, há também diferenças na lógica e organização do trabalho entre as categorias dos trabalhadores da enfermagem^(9)^. O enfermeiro realiza o trabalho assistencial-gerencial e os auxiliares e técnicos em enfermagem o trabalho técnico-assistencial, demarcando a divisão técnica e social do trabalho. Assim, distintos processos de trabalho podem oportunizar diferentes tipos de exposição ao adoecimento^(10)^. Desse modo, não se pode atribuir a infecção apenas a um agente etiológico sem considerar os múltiplos fatores que a influenciam^(11)^ e a heterogeneidade da força de trabalho em saúde.

O exposto evidencia que alguns fatores podem aumentar a susceptibilidade de trabalhadores da enfermagem a infecção pelo SARS-CoV-2 e que a análise da contaminação requer considerar o conjunto de interações sinérgicas entre as questões ocupacionais e sociodemográficas que aumentam a exposição a infecção e ao adoecimento. A análise dos fatores de risco individuais ou laborais é necessária para entender a dinâmica da transmissibilidade, adoecimento e morte pela infecção e para direcionar intervenções rápidas e assertivas. Nesta direção, a natureza socioeconômica da COVID-19 reposiciona os aspectos sociais na análise da crise, uma vez que para o enfrentamento da infecção são essenciais políticas e programas que revertam disparidades sociais, sobretudo no contexto de atuação dos trabalhadores da enfermagem no Sistema Único de Saúde (SUS)^(12)^.

No entanto, a análise de fatores sociodemográficos e ocupacionais associados à infecção pelo SARS-CoV-2 é incipiente na literatura científica para trabalhadores da enfermagem, em contraponto aos estudos que indicam condições associadas e que podem potencializar o adoecimento em outros profissionais da área da saúde^(13)^. Conhecidos os fatores que podem estar associados a infecção pela COVID-19, esses poderão ser mitigados e analisados para que se fortaleçam as políticas públicas e as intervenções para a prevenção de surtos nosocomiais e a proteção dos trabalhadores da enfermagem^(14)^.

É objetivo fundamental da saúde pública garantir não apenas a capacidade do sistema de saúde de fornecer serviços ininterruptos à população afetada, mas também prevenir a transmissão da infecção entre profissionais da saúde, aqui incluídos os trabalhadores da enfermagem, evitando-se o desgaste físico e mental, além da infecção cruzada entre colegas e familiares^(15)^. Além disso, as infecções entre esses trabalhadores reduzem a força de trabalho, aumentam a carga de trabalho daqueles que se mantêm ativos e contribuem para fragilizar o atendimento aos pacientes.

Com base no exposto, o presente estudo objetivou investigar a associação de variáveis sociodemográficas e ocupacionais com a positividade do teste para o SARS-CoV-2 em trabalhadores da enfermagem.

## MÉTODO

### Tipo e Local de Estudo

Trata-se de um estudo exploratório e analítico realizado na rede de instituições de assistência e de administração direta e indireta da Secretaria de Saúde do Estado da Bahia (SESAB) vinculada ao SUS.

### População do Estudo e Critérios de Elegibilidade

A população foi composta por 15.936 trabalhadores de enfermagem (4.852 enfermeiros e 11.084 técnicos e auxiliares em enfermagem) da rede do Sistema Único de Saúde da SESAB, os quais foram monitorados quanto à presença de infecção pelo SARS-CoV-2, no período de março a outubro de 2020.

### Aspectos Éticos

O estudo foi aprovado pelo Comitê de Ética da SESAB, sob parecer número 5.380.246 e pelo Comitê de Ética da Escola de Enfermagem da Universidade Federal da Bahia, parecer número 4.767.147.

### Fonte dos Dados

Utilizou-se um banco de dados secundário disponibilizado pela SESAB, construído por meio do monitoramento dos trabalhadores de enfermagem a partir de março de 2020. O banco foi composto por variáveis sociodemográficas, clínicas, ocupacionais, realização e resultados dos testes para COVID-19, além de dados sobre ocorrência de óbito e internamento desses trabalhadores.

### Fluxo de Monitoramento dos Trabalhadores de Enfermagem da Sesab

O monitoramento dos trabalhadores da enfermagem foi realizado por profissionais da SESAB que integram a Superintendência de Recursos Humanos e a Diretoria de Gestão do Trabalho e Educação na Saúde (DGTES). Disponibilizou-se um questionário às unidades de gestão administrativa, unidades de gestão direta e indireta, das parcerias público-privadas e consórcios públicos interfederativos e dos serviços de saúde ocupacional sob gestão indireta do Estado, aos profissionais de referência e do serviço de recursos humanos de cada unidade de saúde e dos locais onde não havia o Serviço Integrado de Atenção à Saúde do Trabalhador (SIAST) ou Serviço de Segurança e Monitoramento do Trabalho.

O SIAST e o Setor de Saúde Ocupacional e Recursos Humanos de cada unidade ficaram responsáveis por identificar os trabalhadores com suspeita da infecção pelo SARS-CoV-2 (presença de sinais e sintomas e/ou contato com caso confirmado); encaminhar os trabalhadores para realizar a testagem por meio dos testes de reação em cadeia de polimerase com transcriptase reversa (RT-PCR) e/ou teste de antígeno (teste rápido); registrar as informações no prontuário do trabalhador e preencher a planilha de controle de casos suspeitos e/ou confirmados da COVID-19. Os dados obtidos eram enviados à Coordenação de Saúde e Segurança do Trabalhador da Saúde via e-mail para sistematização e tabulação no Excel.

O fluxo de monitoramento dos trabalhadores de enfermagem e registro dos dados está ilustrado na Figura 1.

**Figura 1:** Fluxo de monitoramento dos trabalhadores da enfermagem da Secretaria de Saúde do Estado da Bahia, Salvador, Bahia, Brasil, 2023.

### Variáveis do Estudo

A variável dependente do estudo foi: (0) Infecção não confirmada pelos testes RT-PCR e/ou teste de antígeno e (1) Infecção confirmada pelos testes RT-PCR e/ou teste de antígeno.

As variáveis independentes foram de natureza sociodemográfica e ocupacional. As sociodemográficas incluíram: sexo - (0) feminino, (1) masculino; idade - (0) ≥ 60 anos, (1) 31 a 59 anos e (2) 18 a 30 anos; e raça/cor - (0) branca, amarela e indígena, (1) parda e (2) preta. As variáveis ocupacionais, relacionaram-se a: categoria profissional - (0) enfermeiros (1) técnicos/auxiliares em enfermagem; tipo de vínculo de trabalho - (0) estatutário (1) terceirizado; número de vínculos - (0) Um (1) > 1; setor de trabalho - (0) gerencial (1) assistencial; e contato com caso confirmado para COVID-19 - (0) não (1) sim.

### Análise de Dados

Os dados foram tabulados no *Microsoft Excel* e transportados para o *Statistical Package for Social Science* (SPSS), versão 21. Nas análises descritivas utilizou-se frequências absolutas e percentuais, médias e desvio padrão. Nas análises bivariadas, para verificar a associação entre as variáveis independentes e o desfecho, foram utilizados os testes Qui-quadrado de Pearson e a razão de detecção (RD) e seu respectivo intervalo de confiança de 95%.

As variáveis cujas associações mostraram p-valor ≤ 0,20 foram levadas para a análise multivariada utilizando-se o Modelo de Regressão de Poisson com variância robusta. Foi adotado o procedimento *backward* e o Critério de Informação de Akaike (AIC) para a escolha do melhor modelo. A multicolinearidade foi descartada pela análise do fator de inflação de variância médio das variáveis individualmente, considerando-se valor menor que 10. A significância estatística adotada nessa fase foi de 5%. Os dados foram analisados no *Software* Stata, versão 15.

## RESULTADOS

Dos 15.936 trabalhadores da enfermagem monitorados, 4.057 (25,5%) tiveram diagnóstico laboratorial confirmado para a infecção pelo SARS-CoV-2 (Tabela 1).

**Tabela 1:** Associação entre as variáveis sociodemográficas e ocupacionais e a infecção pelo SARS-CoV-2 para trabalhadores da enfermagem. Salvador, BA, Brasil, 2023.

Variáveis	Total (%)	Diagnóstico laboratorial confirmado para SARS-CoV-2			
		Proporção (%)	* *p-value*	^†^ *RD*	^‡^ *IC95%*
**Sociodemográficas**					
Faixa etária (n = 14.206)					
≥ 60	423(3,0)	74(17,5)	**0,001**	1,00	–
31 a 59	11.405(80,3)	2.894(25,4)		1,45	1,18–1,79
18 a 30	2.378(16,7)	616(25,9)		1,48	1,19–1,84
Sexo (n = 15.936)					
Feminino	13.908(87,3)	3.513(25,3)	0,131	1,00	–
Masculino	2.028(12,7)	544(26,8)		1,06	0,98–1,15
Raça/cor autodeclarada (n = 11.523)					
Branca, amarela e indígena	1.787(15,5)	402(22,5)	**0,030**	1,00	–
Parda	7.310(63,4)	1.691(23,1)		1,03	0,93–1,13
Preta	2.426(21,1)	619(25,5)		1,13	1,02–1,26
**Ocupacionais**					
Categoria profissional (n = 15.936)					
Enfermeiro	4.852(30,4)	1.215(25,0)	0,424	1,00	–
Técnico e/ou auxiliar em enfermagem	11.084(69,6)	2.842(25,6)		1,02	0,97–1,08
Tipo de vínculo de trabalho (n = 14.317)					
Terceirizado	10.030(70,1)	2.644(26,4)	0,248	1,00	–
Estatutário	4.287(29,9)	1.170(27,3)		0,96	0,91–1,02
Nº de vínculos de trabalho (n = 15.890)					
1	12.427(78,2)	2.999(24,1)	**0,000**	1,00	–
> 1	3.463(21,8)	1.055(30,5)		1,26	1,19–1,34
Área de atuação no trabalho (n = 12.287)					
Gerencial	603(4,9)	15(24,9)	0,166	1,00	–
Assistencial	11.684(95,1)	3.208(27,5)		1,10	0,96–1,27
Contato com pessoas com COVID-19 (n = 15.936)					
Não	9.990(62,7)	2.479(24,8)	**0,016**	1,00	–
Sim	5.946(37,3)	1.578(26,5)		1,07	1,01–1,13

*p-value = p-valor;

^†^RD = razão de detecção;

^‡^IC = intervalo de confiança de 95%.

No que se refere as características sociodemográficas predominaram o sexo feminino (87,3%), a faixa etária de 31 a 59 anos (80,3%), sendo a média da idade de 40,2 anos (dp = 9,9), idade mínima de 18 anos e máxima de 79 anos. No quesito raça/cor autodeclarada predominaram trabalhadores pardos (63,4%) e pretos (21,1%) (Tabela 1).

Referente as variáveis do contexto de trabalho, houve maior percentual da categoria de técnicos e/ou auxiliares em enfermagem (69,6%). A maioria referiu vínculo de trabalho terceirizado (70,1%) e aproximadamente um quarto (21,8%) tinha mais de um vínculo. Predominaram trabalhadores que exerciam a função na área assistencial (95,1%) e mais de um terço (37,3%) relataram contato prévio com pessoas diagnosticadas com a COVID-19 (Tabela 1).

Nas análises bivariadas, constatou-se maior proporção da infecção para homens (p = 0,131) e na faixa etária de 18 a 30 anos e de 31 a 59 anos (p-valor = 0,001). A razão de detecção da infecção aumentou em 48% e 45%, respectivamente, para trabalhadores entre 18 a 30 anos (RD = 1,48; IC95% = 1,19–1,84) e 31 a 59 anos (RD = 1,45; IC95% = 1,18–1,79) comparados àqueles com 60 anos ou mais. Os trabalhadores autodeclarados da raça/cor preta foram mais acometidos pela infecção (p-valor = 0,017), observando-se um aumento de 13,0% na razão de detecção para autodeclarados pretos comparados a brancos, amarelos e indígenas (RD = 1,13; IC95% = 1,02–1,26) (Tabela 1).

Relativo aos fatores ocupacionais, ter tido contato com pessoas diagnosticadas com a COVID-19 foi associado a infecção (p-valor = 0,016), observando-se aumento de 1,0% na razão de detecção (RD = 1,07; IC95% = 1,01–1,13). Além disso, a proporção de infecção foi maior para trabalhadores com mais de um vínculo de trabalho (p-valor = ≤ 0,001), notando-se um aumento de 26% (RD = 1,26; IC95% = 1,19–1,34) na razão de detecção. Não houve associação estatisticamente significante a 5,0% entre tipo de vínculo, categoria profissional e área de atuação no trabalho (Tabela 1).

A Tabela 2 apresenta a análise multivariada, na qual as variáveis sexo, faixa etária, raça-cor, número de vínculos, área de atuação e contato com pessoas com COVID-19 foram analisadas concomitantemente. A razão de detecção da infecção foi 33,0% maior na faixa etária de 31 a 59 anos (RD = 1,33; IC95% = 1,05–1,68) e 44% maior na faixa etária de 18 a 30 anos (RD = 1,44; IC95% = 1,12–1,84) quando comparadas a de 60 ou mais anos. A razão de detecção aumentou em 15% nos trabalhadores da raça-cor negra comparados àqueles da raça/cor branca, amarela e indígena (RD = 1,15; IC95% = 1,02–1,28). Ter mais que um vínculo de trabalho aumentou em 23,0% a razão de detecção da infecção (RD = 1,23; IC95% = 1,14–1,33), assim como ter tido contato prévio com pessoas com COVID-19 aumentou em 11,0% a razão de detecção (RD = 1,11; IC95% = 1,03–1,19). Na análise múltipla, escolheu-se o modelo com menor valor do Critério de Informação de Akaike (AIC = 1.1435) (Tabela 2).

**Tabela 2:** Variáveis associadas a infecção pelo SARS-CoV-2 para trabalhadores da enfermagem na análise multivariada. Salvador, BA, Brasil, 2023.

Variáveis	*RD	^†^IC 95%
Faixa etária		
≥ 60 anos		
31 a 59 anos	1,33	1,05-1,68
18 a 30 anos	1,44	1,12-1,84
Raça/cor autodeclarada		
Brancas, amarelas e indígenas		
Pardas	1,05	0,95-1,16
Pretas	1,15	1,02-1,28
N° de vínculos de trabalho		
Um		
> 1	1,23	1,14-1,33
Contato com pessoas com COVID-19		
Não		
Sim	1,11	1,03-1,19
**AIC**	1.1435	

*RD = razão de detecção;

^†^IC = intervalo de confiança de 95%.

## DISCUSSÃO

Nessa investigação, um percentual expressivo de trabalhadores da enfermagem foi infectado pelo SARS-CoV-2. No mesmo período analisado, ao avaliar os trabalhadores de diferentes áreas da Secretaria do Estado da Bahia, observou-se maior proporção da infecção em trabalhadores da enfermagem em comparação a fisioterapeutas (18,0%), farmacêuticos/bioquímicos (16,3%), médicos (15,8%), psicólogos (14,4%), nutricionistas (14,0%) e odontólogos (8,9%). Entre as categorias de nível técnico, as maiores proporções de casos positivos ocorreram em técnicos e auxiliares em enfermagem (22,5%) seguidos por técnicos/auxiliares de laboratório ou patologia (22,4%)^(16)^.

Outros estudos confirmaram maior incidência da infecção em trabalhadores da área da enfermagem. Em uma pesquisa com 4.854 profissionais da saúde iranianos, a incidência de infecção foi maior em enfermeiros (51,3%) em comparação com profissionais de laboratório (3,7%), obstetras (3,0%), técnicos em radiologia (3,0%) e fisioterapeutas (0,4%)^(17)^. Em Wuhan, na China, entre os 2.457 profissionais investigados a maior incidência recaiu sobre os enfermeiros (52,1%), seguidos de médicos (33,6%) e farmacêuticos, técnicos de laboratório e da radiologia (14,3%). Além disso, a maior taxa de infecção foi identificada entre profissionais da saúde (2,10%) em comparação aos profissionais de outras áreas (0,43%)^(18)^.

Os enfermeiros, em geral, passam mais tempo em contato direto com os pacientes do que outros profissionais da saúde. Durante a pandemia, estiveram na linha de frente no combate ao novo coronavírus, evidenciando que, devido às marcas sociais e ao histórico de cuidados, determinados corpos estiveram mais expostos e foram mais exigidos, o que os tornou mais vulneráveis ao impacto da crise sanitária^(17)^.

Corroborando o fato de que nem sempre é possível determinar o trabalho como a origem da contaminação, mesmo com o contato direto dos profissionais com pacientes suspeitos ou diagnosticados com a COVID-19, este estudo demonstrou que variáveis sociodemográficas e ocupacionais foram associadas à infecção, evidenciando sua influência nos indicadores da doença. Os trabalhadores da enfermagem estavam majoritariamente na fase adulta, constatando-se, no modelo multivariado, maior razão de detecção da infecção em estratos etários mais jovens. Similarmente, estudo realizado no Sudoeste do Irã, com 4.854 profissionais da saúde mostrou que a maior incidência da infecção recaiu em trabalhadores da saúde mais jovens, com menos de 25 anos e entre 25 e 45 anos, em comparação à população em geral, e que os enfermeiros foram os mais acometidos^(17)^.

Pesquisas apontam que a infecção pelo SARS-CoV-2 em trabalhadores da enfermagem mais jovens pode estar associada a inexperiência laboral, à falta de treinamento para a utilização de equipamentos de proteção individual (EPI) e para o manejo de pacientes críticos, o que pode potencializar a cadeia de transmissão viral^(19,20)^. É possível que a maior idade esteja relacionada ao aumento do conhecimento e a maior experiência, favorecendo mais prudência nas medidas de prevenção da transmissão de infecções e no uso adequado de EPIs^(21)^.

Nesse estudo, a razão de detecção da infecção pelo SARS-CoV-2 foi maior em trabalhadores autodeclarados pretos, corroborando uma investigação realizada com 14.441 profissionais da saúde do Serviço Nacional de Saúde da Inglaterra, cuja maior taxa de contaminação recaiu em profissionais de etnia BAME (negros, asiáticos, ou qualquer outra minoria étnica) em comparação aos de etnia branca^(22)^. Os autores consideraram que as taxas mais altas em profissionais da saúde de origem BAME podem estar ligadas a piores indicadores econômicos e sociais^(22)^. Nesse cenário, a vigilância aprimorada e as medidas de controle da infecção devem ser prioridade nesses grupos sociais^(18)^.

De acordo com o relatório sobre o perfil sociorracial da enfermagem no Brasil, as mulheres negras representam 53,0% dos trabalhadores da enfermagem no país. Embora haja maior percentual de técnicos e auxiliares em enfermagem negros (40%) do que enfermeiros (32,5%), ambas categorias tendem a ocupar postos de trabalho mais precarizados, enfrentam riscos laborais diversos e lidam com a escassez de EPIs^(23,24)^. O fato de trabalhadores da enfermagem negros comporem o grupo mais exposto durante a pandemia coaduna-se com vulnerabilidades históricas da profissão demarcadas pela sinergia de marcadores sociais de gênero, classe social e raça-cor.

A infecção pelo SARS-CoV-2 foi também associada a ter mais de um vínculo de trabalho, corroborando pesquisa realizada com 415 profissionais da enfermagem de um hospital de ensino, na cidade de São Paulo, o qual mostrou 2,27 vezes mais chance de infecção para aqueles que atuavam em mais de um serviço de saúde^(25)^. Muitos trabalhadores da enfermagem assumem mais de um posto de trabalho, principalmente devido a baixos salários e vínculos precários^(26)^. Quanto maior o número de vínculos, maior é o tempo de exposição aos fatores de riscos para a COVID-19, a exemplo do aumento da probabilidade de contato com pacientes e colegas de trabalho contaminados, o que torna os trabalhadores mais vulneráveis a infecção e ao adoecimento físico e mental^(7,13,14)^.

É importante destacar que a maior carga viral à qual os trabalhadores estão expostos no ambiente de trabalho pode influenciar o risco de contaminação. Um estudo ecológico brasileiro, com 7.201 profissionais da área da enfermagem com COVID-19, evidenciou maior carga viral no ambiente hospitalar, o que aumentava o risco de infecção para esses profissionais. Utilizou-se como parâmetro a Carga de Doença Global (GBD) estimada em 0,051 (0,032-0,074), a qual estava em tendência de alta, com maior impacto nos mais jovens e no sexo feminino^(26)^.

Outro fator associado à infecção foi ter contato com caso confirmado, reforçando que é fundamental a disponibilização e o uso adequado de EPIs aos trabalhadores da enfermagem. Estudos nacionais e internacionais também constataram maior proporção de testes positivos para o SARS-CoV-2 em profissionais da saúde que tiveram contato direto com pacientes, familiares ou colegas de trabalho infectados^(5,15,27,28)^.

No presente estudo, não houve associação entre a natureza do trabalho, seja assistencial ou gerencial, e a infecção pelo SARS-CoV-2. Da mesma forma, não houve associação entre a categoria profissional (técnicos/auxiliares em enfermagem ou enfermeiros) e a infecção. Em outra investigação, foi observada uma taxa de infecção de 15% entre os profissionais envolvidos diretamente no atendimento ao paciente, 16% entre aqueles que não estavam diretamente envolvidos, mas trabalhavam em áreas de alto risco, como laboratórios, e 18% entre trabalhadores não clínicos. Assim, a participação no atendimento direto ao paciente não foi considerada um fator de risco para a positividade no teste RT-PCR para SARS-CoV-2^(29)^. Outras pesquisas também constataram que a exposição ao risco de infecção foi maior entre os funcionários administrativos, sugerindo que esses profissionais podem subestimar o risco de contaminação por não estarem diretamente envolvidos no cuidado aos pacientes, o que frequentemente leva à não adesão ao uso de máscaras. Uma possível explicação para isso é a menor conscientização de que as infecções também podem ser transmitidas pelos colegas de trabalho ^(21,30)^.

Do ponto de vista dos fatores ocupacionais e da segurança do trabalho em saúde, a investigação revelou a necessidade de investimento em ações promotoras da saúde ocupacional, assegurando proteção individual e coletiva no ambiente de trabalho. Revelou, também, que é fundamental ser dada atenção à capacitação profissional para atuação em contextos de pandemia, especialmente com foco nos profissionais mais jovens e menos experientes, assim como salienta a necessidade de se considerar marcadores sociais de risco para o adoecimento, como maior número de vínculos e raça-cor negra, nas estratégias de prevenção e controle da infecção. O estudo mostrou a necessidade da valorização econômica do trabalho no campo da enfermagem para minimizar a precarização do trabalho e permitir a redução do número de vínculos. É possível, diante da associação entre os fatores analisados, considerar a infecção pelo SARS-CoV-2 como um incidente e/ou agravo também relacionado ao trabalho^(7,20)^.

Este estudo apresenta algumas limitações. Primeiramente, foi realizado com um grupo de trabalhadores da enfermagem de um único estado do Brasil. Em segundo lugar, o número de casos confirmados foi reunido durante o período de monitoramento dos trabalhadores da SESAB, não abrangendo toda a duração da pandemia, o que pode ter levado à subestimação das taxas de infecção. Por fim, possíveis fatores relacionados aos indicadores de infecção pelo SARS-CoV-2, como medidas de higiene pessoal e uso de EPIs, não foram considerados.

No entanto, os resultados deste estudo evidenciaram e reforçaram fatores de risco sociais e ocupacionais que demandam respostas eficazes de políticas públicas contra a COVID-19, além de ações voltadas à valorização do trabalho em enfermagem. O estudo também destacou a necessidade de apoio e alerta para trabalhadores mais jovens, com múltiplos vínculos de trabalho e autodeclarados negros, que enfrentam maior risco de infecção. Além disso, reforçou a importância da disponibilização de EPIs de qualidade e da adoção rigorosa de medidas protetivas. Dessa forma, este estudo oferece lições valiosas para a gestão de novas crises sanitárias.

## CONCLUSÃO

Um quantitativo expressivo de trabalhadores da área da enfermagem teve o diagnóstico laboratorial confirmado para o SARS-CoV-2. As variáveis que mais contribuíram para a infeção foram faixa etária entre 18 e 59 anos, raça/cor preta, ter mais de um vínculo de trabalho e ter contato com pessoas infectadas, mostrando a sua influência nos indicadores da COVID-19. Essas variáveis salientaram a importância de políticas públicas e intervenções direcionadas ao apoio dos trabalhadores, a promoção de um ambiente de trabalho seguro e a valorização econômica do trabalho em enfermagem, o que envolve assegurar salários dignos e compatíveis com suas responsabilidades.

## Data Availability

O conjunto de dados que dá suporte aos resultados deste estudo não está disponível publicamente.

# Translation: Factores asociados a la positividad del SARS-COV-2 en trabajadores de enfermería

**DOI:** 10.1590/1980-220X-REEUSP-2024-0301es

**Authors:** Jones Sidnei Barbosa de Oliveira, Fernanda Carneiro Mussi, Ana Carla Carvalho Coelho, Jules Ramon Brito Teixeira, Tatiane Araújo dos Santos, Angélica Araújo de Menezes, Luciano de Paula Moura

**Affiliations:** 1 Universidade Federal da Bahia, Salvador, BA, Brasil.

**Keywords:** Enfermería, Infecciones por Coronavírus, COVID-19, Pandemias, Vigilancia de la Salud del Trabajador

## Abstract

**Objetivo:**

Investigar la asociación de variables sociodemográficas y laborales con la positividad al SARS-CoV-2 en trabajadores de enfermería.

**Método:**

Estudio exploratorio y analítico realizado con una población de 15.936 trabajadores. Se utilizó una base de datos secundaria y los datos se analizaron mediante estadística descriptiva e inferencial, utilizando el Modelo de Regresión de Poisson robusto y significación estadística del 5%.

**Resultados:**

Hubo un predominio de mujeres (87,3%), de 31 a 59 años (80,3%), de raza/color moreno (63,4%), de técnicos/auxiliares de enfermería (69,6%), de cuidadores (95,1%), de trabajadores subcontratados (70,1%) y de trabajadores solteros (78,2%). El rango de edad de 31 a 59 años (IC 95%: 1,05–1,68) y de 18 a 30 años (IC 95%: 1,12–1,84), la raza/color negro (IC 95%: 1,02–1,28), tener más de un trabajo (IC 95%: 1,14 –1,33) y tener contacto con un caso confirmado (IC 95%: 1,03–1,19) se asociaron con la infección en el análisis multivariante.

**Conclusión:**

La detección de la infección se asoció a trabajadores adultos, de raza negra, con más de una relación laboral y contacto con personas infectadas por SARS-CoV-2.

## INTRODUCCIÓN

La pandemia del síndrome respiratorio agudo grave por coronavirus 2 (SARS-CoV-2) se ha propagado rápidamente, lo que ha llevado a la Organización Mundial de la Salud a declarar una emergencia de salud pública de importancia internacional^(1)^. Hasta marzo de 2023, se habían confirmado en todo el mundo más de 760.360.956 casos de COVID-19 y 6.873.477 muertes. En este periodo, Brasil ocupaba el 6º lugar entre los países con mayor número de casos (37.145.514) y el 2º en cuanto a muertes (699.634), con una tasa de letalidad del 1,9%^(2)^.

Entre la población afectada por la infección, los trabajadores de salud se han visto ampliamente afectados, con 146.685 casos de COVID-19 confirmados en Brasil sólo en 2021^(3)^. Según los Centros para el Control y la Prevención de Enfermedades de EE.UU., alrededor del 11% de las infecciones por SARS-CoV-2 han afectado a trabajadores sanitarios^(4)^. Entre ellos, los trabajadores de enfermería registraron el mayor número de casos, con 43.577 entre técnicos y auxiliares de enfermería y 24.719 entre enfermeras. La mayor vulnerabilidad a la infección de estos trabajadores fue tres veces superior a la de la población general^(5)^.

El Consejo Internacional de Enfermeras y la Organización Mundial de la Salud han registrado que hay 28 millones de trabajadores de enfermería en el mundo, destacando la importancia de esta fuerza de trabajo en los sistemas de salud. En Brasil, este grupo representa aproximadamente dos millones de profesionales en todas las estructuras organizativas del sistema de salud y en todos los niveles de atención^(6)^. Por ser la mayor fuerza de trabajo en salud, han estado en la primera línea de la pandemia de COVID-19 y, debido a la exposición al virus, ha aumentado el número de trabajadores infectados que requieren atención hospitalaria^(6)^.

Las variables ocupacionales de estos trabajadores pueden estar asociadas a la infección. Enfermeras, técnicos y auxiliares de enfermería están en contacto con pacientes sospechosos y/o infectados las 24 horas del día, trabajando por turnos. En general, están más expuestos a la fatiga física y al estrés laboral^(7,8)^, y a menudo desempeñan más de un trabajo. Hay que tener en cuenta que el personal de enfermería tiene características específicas relacionadas con la naturaleza del trabajo realizado, marcadores ocupacionales (carga de trabajo, número de trabajos, turnos e intensidad del trabajo), sociales (raza, género, clase social y grupo de edad), políticos y económicos, que pueden ser determinantes de una mayor susceptibilidad a la infección en los servicios de salud^(9)^.

A pesar de las diferencias en el trabajo en salud, también existen diferencias en la lógica y la organización del trabajo entre las categorías de trabajadores de enfermería^(9)^. Las enfermeras realizan un trabajo de gestión de los cuidados y los auxiliares y técnicos de enfermería un trabajo técnico-asistencial, delimitando la división técnica y social del trabajo. Así, los diferentes procesos de trabajo pueden dar lugar a diferentes tipos de exposición a la enfermedad^(10)^. Así, la infección no puede atribuirse a un único agente etiológico sin tener en cuenta los múltiples factores que influyen en ella^(11)^y la heterogeneidad del personal en salud.

Lo anterior demuestra que algunos factores pueden aumentar la susceptibilidad de los trabajadores de enfermería a la infección por SARS-CoV-2 y que el análisis de la contaminación requiere considerar el conjunto de interacciones sinérgicas entre aspectos ocupacionales y sociodemográficos que aumentan la exposición a la infección y la enfermedad. El análisis de los factores de riesgo individuales u ocupacionales es necesario para comprender la dinámica de la transmisibilidad, la enfermedad y la muerte por infección y para dirigir intervenciones rápidas y asertivas. En este sentido, la naturaleza socioeconómica del COVID-19 reposiciona los aspectos sociales en el análisis de la crisis, ya que políticas y programas que reviertan las disparidades sociales son esenciales para enfrentar la infección, especialmente en el contexto de los trabajadores de enfermería del Sistema Único de Salud (SUS)^(12)^.

Sin embargo, el análisis de los factores sociodemográficos y ocupacionales asociados a la infección por SARS-CoV-2 es incipiente en la literatura científica para los trabajadores de enfermería, en contraste con los estudios que indican condiciones asociadas que pueden potenciar la enfermedad en otros profesionales de la salud^(13)^. Una vez que se conozcan los factores que pueden estar asociados a la infección por COVID-19, se podrán mitigar y analizar con el fin de reforzar las políticas e intervenciones públicas para prevenir los brotes nosocomiales y proteger a los trabajadores de enfermería^(14)^.

Es un objetivo fundamental de la salud pública garantizar no sólo la capacidad del sistema de salud para prestar servicios ininterrumpidos a la población afectada, sino también prevenir la transmisión de infecciones entre los profesionales de la salud, incluidos los trabajadores de enfermería, evitando el desgaste físico y mental, así como la infección cruzada entre colegas y familiares^(15)^. Además, las infecciones entre estos trabajadores reducen la plantilla, aumentan la carga de trabajo de los que permanecen en activo y contribuyen a debilitar la atención al paciente.

Basándose en lo anterior, este estudio se propuso investigar la asociación de las variables sociodemográficas y ocupacionales con la positividad a la prueba del SARS-CoV-2 en trabajadores de enfermería.

## MÉTODO

### Tipo y Localización del Estudio

Se trata de un estudio exploratorio y analítico realizado en la red de instituciones asistenciales y de administración directa e indirecta del Departamento de Salud del Estado de Bahía (SESAB) vinculadas al SUS.

### Población de Estudio y Criterios de Elegibilidad

La población estuvo constituida por 15.936 trabajadores de enfermería (4.852 enfermeros y 11.084 técnicos y auxiliares de enfermería) de la red del Sistema Único de Salud (SUS) del SESAB, que fueron monitoreados para detectar la presencia de infección por SARS-CoV-2 entre marzo y octubre de 2020.

### Aspectos Éticos

El estudio fue aprobado por el Comité de Ética de la SESAB, con el número de dictamen 5.380.246, y por el Comité de Ética de la Escuela de Enfermería de la Universidad Federal de Bahía, con el número de dictamen 4.767.147.

### Fuente de Datos

Se utilizó una base de datos secundaria puesta a disposición por el SESAB, construida mediante el seguimiento de los trabajadores de enfermería hasta marzo de 2020. La base de datos estaba compuesta por variables sociodemográficas, clínicas y ocupacionales, realización y resultados de la prueba COVID-19, así como datos sobre la ocurrencia de muerte y hospitalización de estos trabajadores.

### Flujo de Monitoreo de los Trabajadores de Enfermería del Sesab

El monitoreo de los trabajadores de enfermería fue realizado por profesionales del SESAB que forman parte de la Superintendencia de Recursos Humanos y de la Dirección de Gestión del Trabajo y Educación en Salud (DGTES). Se puso un cuestionario a disposición de las unidades de gestión administrativa, unidades de gestión directa e indirecta, asociaciones público-privadas y consorcios públicos interfederativos y servicios de salud ocupacional bajo gestión estatal indirecta, a los profesionales de referencia y de los servicios de recursos humanos en cada unidad de salud y en los lugares donde no había Servicio Integrado de Atención a la Salud de los Trabajadores (SIAST) ni Servicio de Vigilancia y Seguridad en el Trabajo.

El SIAST y el Departamento de Salud Laboral y Recursos Humanos de cada unidad se encargaron de identificar a los trabajadores sospechosos de estar infectados por el SARS-CoV-2 (presencia de signos y síntomas y/o contacto con un caso confirmado); remitir a los trabajadores para la realización de pruebas mediante la reacción en cadena de la polimerasa con transcriptasa inversa (RT-PCR) y/o la prueba de antígenos (prueba rápida); registrar la información en la historia clínica del trabajador y cumplimentar la hoja de control de casos sospechosos y/o confirmados COVID-19. Los datos obtenidos se enviaron a la Coordinación de Salud y Seguridad Laboral por correo electrónico para su sistematización y tabulación en Excel.

El flujo de seguimiento de los trabajadores de enfermería y registro de datos se ilustra en la Figura 1.

**Figura 1:** Flujo de monitorización de los trabajadores de enfermería en el Departamento de Salud del Estado de Bahía, Salvador, Bahía, Brasil, 2023.

### Variables del Estudio

La variable dependiente del estudio fue: (0) Infección no confirmada por RT-PCR y/o pruebas de antígeno y (1) Infección confirmada por RT-PCR y/o pruebas de antígeno.

Las variables independientes fueron sociodemográficas y ocupacionales. Las variables sociodemográficas incluían: sexo - (0) femenino, (1) masculino; edad - (0) ≥ 60 años, (1) 31 a 59 años y (2) 18 a 30 años; y raza/color - (0) blanca, amarilla e indígena, (1) morena y (2) negra. Las variables ocupacionales estaban relacionadas con: categoría profesional - (0) enfermeros (1) técnicos/auxiliares de enfermería; tipo de relación laboral - (0) estatutaria (1) subcontratada; número de relaciones laborales - (0) Una (1) > 1; sector laboral - (0) directivo (1) asistencial; y contacto con un caso confirmado de COVID-19 - (0) no (1) sí.

### Análisis de los Datos

Los datos se tabularon en*Microsoft* *Excel*y se transfirieron al*paquete estadístico Statistical Package for Social Science* (SPSS), versión 21. En los análisis descriptivos se utilizaron frecuencias absolutas y porcentuales, medias y desviación estándar. En los análisis bivariados se utilizaron la prueba de chi cuadrado de Pearson y la razón de detección (RD) y su respectivo intervalo de confianza del 95% para comprobar la asociación entre las variables independientes y el resultado. Las variables cuyas asociaciones mostraron un valor p ≤ 0,20 se llevaron al análisis multivariante mediante el modelo de regresión de Poisson con varianza robusta. Se utilizó el procedimiento*backward* y el Criterio de Información de Akaike (AIC) para elegir el mejor modelo. La multicolinealidad se descartó analizando el factor de inflación de la varianza media de las variables individuales, considerando un valor inferior a 10. La significación estadística adoptada en esta fase fue del 5%. Los datos se analizaron con el*programa*Stata, versión 15.

## RESULTADOS

De los 15.936 trabajadores de enfermería monitorizados, 4.057 (25,5%) tenían diagnóstico de laboratorio confirmado de infección por SARS-CoV-2 (Tabla 1).

**Tabla 1:** Asociación entre variables sociodemográficas y ocupacionales y la infección por SARS-CoV-2 en trabajadores de enfermería – Salvador, BA, Brasil, 2023.

Variables	Total (%)	Diagnóstico laboratorio confirmado para SARS-CoV-2			
		Proporción (%)	* *p-valor*	^†^ *RD*	^‡^ *IC95%*
**Sociodemográficas**					
Grupos etarios (n = 14.206)					
≥ 60	423(3,0)	74(17,5)	**0,001**	1,00	–
31 a 59	11.405(80,3)	2.894(25,4)		1,45	1,18–1,79
18 a 30	2.378(16,7)	616(25,9)		1,48	1,19–1,84
Sexo (n = 15.936)					
Femenino	13.908(87,3)	3.513(25,3)	0,131	1,00	–
Masculino	2.028(12,7)	544(26,8)		1,06	0,98–1,15
Raza/color autodeclarada (n = 11.523)					
Blanca, amarilla e indígena	1.787(15,5)	402(22,5)	**0,030**	1,00	–
Parda	7.310(63,4)	1.691(23,1)		1,03	0,93–1,13
Negra	2.426(21,1)	619(25,5)		1,13	1,02–1,26
**Ocupacionales**					
Categoría profesional (n = 15.936)					
Enfermero	4.852(30,4)	1.215(25,0)	0,424	1,00	–
Técnico y/o auxiliar de enfermería	11.084(69,6)	2.842(25,6)		1,02	0,97–1,08
Tipo de vínculo de trabajo (n = 14.317)					
Tercerizado	10.030(70,1)	2.644(26,4)	0,248	1,00	–
Estatutario	4.287(29,9)	1.170(27,3)		0,96	0,91–1,02
Nº de vínculos de trabajo (n = 15.890)					
1	12.427(78,2)	2.999(24,1)	**0,000**	1,00	–
> 1	3.463(21,8)	1.055(30,5)		1,26	1,19–1,34
Área de actuación en el trabajo (n = 12.287)					
Gerencial	603(4,9)	15(24,9)	0,166	1,00	–
Asistencial	11.684(95,1)	3.208(27,5)		1,10	0,96–1,27
Contacto con personas con COVID-19 (n = 15.936)					
No	9.990(62,7)	2.479(24,8)	**0,016**	1,00	–
Si	5.946(37,3)	1.578(26,5)		1,07	1,01–1,13

*p-valor;

^†^RD = razón de detección;

^‡^IC = intervalo de confianza de 95%.

En cuanto a las características sociodemográficas, la mayoría eran mujeres (87,3%), con edades comprendidas entre los 31 y los 59 años (80,3%), con una edad media de 40,2 años (DE = 9,9), una edad mínima de 18 años y una máxima de 79 años. En cuanto a la raza/color autodeclarada, predominaban los trabajadores pardos (63,4%) y negros (21,1%) (Tabla 1).

En cuanto a las variables de contexto laboral, hubo un mayor porcentaje de técnicos y/o auxiliares de enfermería (69,6%). La mayoría declaró tener un trabajo externalizado (70,1%) y aproximadamente una cuarta parte (21,8%) tenía más de un trabajo. Predominaron los cuidadores (95,1%) y más de un tercio (37,3%) refirió contacto previo con personas diagnosticadas de COVID-19 (Tabla 1).

Los análisis bivariados mostraron una mayor proporción de infección en hombres (p = 0,131) y en los grupos de edad de 18–30 y 31–59 años (valor p = 0,001). La proporción de detección de la infección aumentó un 48% y un 45%, respectivamente, en los trabajadores de 18 a 30 años (DR = 1,48; IC95% = 1,19–1,84) y de 31 a 59 años (RD = 1,45; IC95% = 1,18–1,79) en comparación con los de 60 años o más. Los trabajadores autodeclarados de raza/color negra se vieron más afectados por la infección (p-valor = 0,017), con un aumento del 13,0% en la razón de detección para los negros autodeclarados en comparación con los blancos, amarillos e indígenas (RD = 1,13; IC 95% = 1,02–1,26) (Tabla 1).

En cuanto a los factores ocupacionales, haber tenido contacto con personas diagnosticadas de COVID-19 se asoció con la infección (p-valor = 0,016), con un aumento del 1,0% en la proporción de detección (RD = 1,07; IC 95% = 1,01–1,13). Además, la proporción de infección fue mayor en los trabajadores con más de una relación laboral (p-valor = ≤ 0,001), con un aumento del 26% (RD = 1,26; IC 95% = 1,19–1,34) en la razón de detección. No hubo asociación estadísticamente significativa al 5,0% entre tipo de empleo, categoría profesional y área de trabajo (Tabla 1).

La Tabla 2 muestra el análisis multivariante, en el que se analizaron concomitantemente las variables sexo, grupo de edad, raza-color, número de trabajos, área de trabajo y contacto con personas con COVID-19. La tasa de detección de la infección fue un 33,0% superior en el grupo de edad de 31 a 59 años (RD = 1,33; IC95% = 1,05–1,68) y un 44% superior en el grupo de edad de 18 a 30 años (RD = 1,44; IC95% = 1,12–1,84) en comparación con el grupo de edad de 60 años o más. La tasa de detección aumentó un 15% en los trabajadores negros en comparación con los blancos, amarillos e indígenas (RD = 1,15; IC 95% = 1,02-1,28). Tener más de una relación laboral aumentó la tasa de detección de la infección en un 23,0% (RD = 1,23; IC 95% = 1,14–1,33), mientras que haber tenido contacto previo con personas con COVID-19 aumentó la tasa de detección en un 11,0% (RD = 1,11; IC 95% = 1,03–1,19). En el análisis múltiple, se eligió el modelo con el valor más bajo del Criterio de Información de Akaike (AIC = 1,1435) (Tabla 2).

**Tabla 2:** Variables asociadas a la infección por SARS-CoV-2 en trabajadoras de enfermería en el análisis multivariante – Salvador, BA, Brasil, 2023.

Variables	*RD	^†^IC 95%
Grupos etarios		
≥ 60 años		
31 a 59 años	1,33	1,05–1,68
18 a 30 años	1,44	1,12–1,84
Raza/color autodeclarada		
Blanca, amarilla e indígena		
Parda	1,05	0,95–1,16
Negra	1,15	1,02–1,28
N° de vínculos de trabajo		
Uno		
> 1	1,23	1,14–1,33
Contacto con personas con COVID-19		
No		
Si	1,11	1,03–1,19
**AIC**	1.1435	

*RD = razón de detección;

^†^IC = intervalo de confianza de 95%.

## DISCUSIÓN

En esta investigación, un porcentaje significativo de trabajadores de enfermería se infectó con SARS-CoV-2. En el mismo período analizado, al evaluar trabajadores de diferentes áreas de la Secretaría del Estado de Bahía, se observó una mayor proporción de infección en trabajadores de enfermería en comparación con fisioterapeutas (18,0%), farmacéuticos/bioquímicos (16,3%), médicos (15,8%), psicólogos (14,4%), nutricionistas (14,0%) y odontólogos (8,9%). Entre las categorías de nivel técnico, las proporciones más elevadas de casos positivos se dieron en técnicos y auxiliares de enfermería (22,5%), seguidos de técnicos/asistentes de laboratorio o patología (22,4%)^(16)^.

Otros estudios han confirmado una mayor incidencia de la infección entre los trabajadores de enfermería. En una encuesta realizada a 4.854 trabajadores sanitarios iraníes, la incidencia de infección fue mayor entre los enfermeros (51,3%) que entre los trabajadores de laboratorio (3,7%), los obstetras (3,0%), los técnicos de radiología (3,0%) y los fisioterapeutas (0,4%)^(17)^. En Wuhan (China), entre los 2.457 profesionales investigados, la mayor incidencia se registró entre los enfermeros (52,1%), seguidos de los médicos (33,6%) y los farmacéuticos y técnicos de laboratorio y radiología (14,3%). Además, la mayor tasa de infección se identificó entre los profesionales de salud (2,10%) en comparación con los profesionales de otras áreas (0,43%)^(18)^.

Por lo general, los enfermeros pasan más tiempo en contacto directo con los pacientes que otros profesionales sanitarios. Durante la pandemia, han estado en primera línea en la lucha contra el nuevo coronavirus, lo que demuestra que, debido a las marcas sociales y a la historia de los cuidados, ciertos cuerpos han estado más expuestos y han sido más exigidos, lo que les ha hecho más vulnerables al impacto de la crisis sanitaria^(17)^.

Corroborando el hecho de que no siempre es posible determinar el trabajo como origen de la contaminación, incluso con el contacto directo de los profesionales con pacientes sospechosos o diagnosticados de COVID-19, este estudio mostró que las variables sociodemográficas y ocupacionales estaban asociadas a la infección, destacando su influencia en los indicadores de la enfermedad. La mayoría de los trabajadores de enfermería eran adultos, y el modelo multivariante mostró una mayor tasa de detección de infecciones en los grupos de edad más jóvenes. De forma similar, un estudio realizado en el suroeste de Irán con 4.854 trabajadores sanitarios mostró que la mayor incidencia de infección se daba entre los trabajadores sanitarios más jóvenes, menores de 25 años y entre 25 y 45 años, en comparación con la población general, y que los enfermeros eran los más afectados^(17)^.

Las investigaciones han demostrado que la infección por SARS-CoV-2 en los trabajadores de enfermería más jóvenes puede estar asociada a la inexperiencia en el trabajo, a la falta de formación en el uso de equipos de protección individual (EPI) y en el manejo de pacientes en estado crítico, lo que puede aumentar la cadena de transmisión vírica^(19,20)^. Es posible que la mayor edad esté relacionada con un mayor conocimiento y experiencia, favoreciendo una mayor prudencia en las medidas para prevenir la transmisión de infecciones y el uso adecuado de EPI^(21)^.

En este estudio, la tasa de detección de la infección por SARS-CoV-2 fue mayor en los trabajadores negros autodeclarados, lo que corrobora una investigación realizada con 14.441 trabajadores sanitarios del Servicio Nacional de Salud de Inglaterra, cuya mayor tasa de contaminación recayó en los trabajadores BAME (negros, asiáticos o de cualquier otra minoría étnica) en comparación con los trabajadores blancos^(22)^. Los autores consideraron que las mayores tasas en trabajadores sanitarios de origen BAME pueden estar relacionadas con peores indicadores económicos y sociales^(22)^. En este escenario, la mejora de las medidas de vigilancia y control de la infección debería priorizarse en estos grupos sociales^(18)^.

Según el informe sobre el perfil socio-racial de la enfermería en Brasil, las mujeres negras representan el 53,0% de los trabajadores de enfermería del país. Aunque haya un porcentaje mayor de técnicos y auxiliares de enfermería negros (40%) que de enfermeros (32,5%), ambas categorías tienden a ocupar puestos de trabajo más precarios, a enfrentarse a diversos riesgos laborales y a lidiar con la escasez de EPI^(23,24)^. El hecho de que los trabajadores de enfermería negros constituyan el grupo más expuesto durante la pandemia está en consonancia con las vulnerabilidades históricas de la profesión, delimitadas por la sinergia de los marcadores sociales de género, clase social y raza-color.

La infección por SARS-CoV-2 también se asoció a tener más de un trabajo, corroborando un estudio realizado con 415 profesionales de enfermería en un hospital universitario de la ciudad de São Paulo, que mostró una probabilidad de infección 2,27 veces mayor para los que trabajaban en más de un servicio de salud^(25)^. Muchos trabajadores de enfermería tienen más de un empleo, principalmente debido a los bajos salarios y a la precariedad de las relaciones laborales^(26)^. Cuanto mayor es el número de empleos, mayor es el tiempo de exposición a factores de riesgo de COVID-19, como la mayor probabilidad de contacto con pacientes y compañeros de trabajo contaminados, lo que hace que los trabajadores sean más vulnerables a las infecciones y a las enfermedades físicas y mentales^(7,13,14)^.

Es importante destacar que la mayor carga viral a la que están expuestos los trabajadores en el lugar de trabajo puede influir en el riesgo de contaminación. Un estudio ecológico brasileño de 7.201 profesionales de enfermería con COVID-19 mostró una mayor carga viral en el entorno hospitalario, lo que aumentó el riesgo de infección para estos profesionales. Se utilizó como parámetro la Carga Global de Enfermedad (CGE) estimada en 0,051 (0,032-0,074), que presentaba una tendencia ascendente, con mayor impacto en las personas más jóvenes y en el sexo femenino^(26)^.

Otro factor asociado a la infección fue haber estado en contacto con un caso confirmado, lo que subraya la importancia de proporcionar y utilizar adecuadamente EPI a los trabajadores de enfermería. Estudios nacionales e internacionales también han encontrado una mayor proporción de pruebas positivas para el SARS-CoV-2 en trabajadores sanitarios que han tenido contacto directo con pacientes infectados, familiares o compañeros de trabajo^(5,15,27,28)^.

En el presente estudio, no hubo asociación entre la naturaleza del trabajo, ya fuera asistencial o de gestión, y la infección por SARS-CoV-2. Del mismo modo, no hubo asociación entre la categoría profesional (técnicos/auxiliares de enfermería o enfermeras) y la infección. En otra investigación, se observó una tasa de infección del 15% entre los profesionales que participaban directamente en la atención al paciente, del 16% entre los que no participaban directamente, pero trabajaban en zonas de alto riesgo, como los laboratorios, y del 18% entre los trabajadores no clínicos. Así pues, la participación en la atención directa al paciente no se consideró un factor de riesgo de positividad en la prueba RT-PCR para el SARS-CoV-2^(29)^. En otras investigaciones también se ha observado que la exposición al riesgo de infección era mayor entre el personal administrativo, lo que sugiere que estos profesionales pueden subestimar el riesgo de contaminación porque no participan directamente en la atención al paciente, lo que a menudo conduce a la no adhesión al uso de mascarillas. Una posible explicación es una menor concienciación de que las infecciones también pueden ser transmitidas por los compañeros de trabajo^(21,30)^.

Desde el punto de vista de los factores ocupacionales y de la seguridad en salud laboral, la investigación reveló la necesidad de invertir en acciones de promoción de la salud laboral, garantizando la protección individual y colectiva en el lugar de trabajo. También revela que es esencial prestar atención a la formación profesional para trabajar en contextos pandémicos, especialmente con foco en los profesionales más jóvenes y con menos experiencia, además de destacar la necesidad de considerar marcadores sociales de riesgo de enfermedad, como el mayor número de corbatas y la raza negra, en las estrategias de prevención y control de infecciones. El estudio puso de manifiesto la necesidad de valorar económicamente el trabajo en el ámbito de la enfermería para minimizar la precariedad laboral y permitir una reducción del número de puestos de trabajo. Dada la asociación entre los factores analizados, es posible considerar la infección por SARS-CoV-2 como un incidente y/o afección relacionada con el trabajo^(7,20)^.

Este estudio tiene algunas limitaciones. En primer lugar, se realizó con un grupo de trabajadores de enfermería de un único estado de Brasil. En segundo lugar, el número de casos confirmados se recogió durante el periodo de seguimiento de los trabajadores del SESAB, y no abarcó toda la duración de la pandemia, lo que puede haber llevado a una subestimación de las tasas de infección. Por último, no se tuvieron en cuenta posibles factores relacionados con los indicadores de infección por SARS-CoV-2, como las medidas de higiene personal y el uso de EPI.

Sin embargo, los resultados de este estudio pusieron de relieve y reforzaron los factores de riesgo social y laboral que exigen respuestas eficaces de las políticas públicas contra el COVID-19, así como acciones encaminadas a valorar el trabajo de enfermería. El estudio también puso de relieve la necesidad de apoyar y advertir a los trabajadores más jóvenes, a los que tienen múltiples relaciones laborales y a los trabajadores negros autodeclarados, que se enfrentan a un mayor riesgo de infección. También reforzó la importancia de proporcionar EPI de calidad y de adoptar rigurosamente medidas de protección. De este modo, este estudio ofrece valiosas lecciones para la gestión de nuevas crisis sanitarias.

## CONCLUSIÓN

Se confirmó el diagnóstico de laboratorio de SARS-CoV-2 en un número significativo de profesionales de enfermería. Las variables que más contribuyeron para la infección fueron edad entre 18 y 59 años, raza/color negro, tener más de una relación laboral y tener contacto con personas infectadas, mostrando su influencia en los indicadores COVID-19. Estas variables resaltan la importancia de políticas públicas e intervenciones dirigidas a apoyar a los trabajadores, promover un ambiente de trabajo seguro y valorar económicamente el trabajo de enfermería, lo que implica garantizar salarios dignos y compatibles con sus responsabilidades.

## Data Availability

El conjunto de datos que respalda los hallazgos de este estudio no está disponible en público.
